# Paraphenylenediamine allergic contact dermatitis in an African American male

**DOI:** 10.1016/j.jdcr.2023.10.014

**Published:** 2023-11-03

**Authors:** Jessica McClain, Amanda Stewart Brown, Caitlin Alexis Noble, Stephen E. Helms, Robert T. Brodell

**Affiliations:** a University of Mississippi Medical Center, Jackson, Mississippi; b Department of Dermatology, University of Mississippi Medical Center, Jackson, Mississippi

**Keywords:** African American, allergic contact dermatitis, paraphenylenediamine, type V skin

## Introduction

Paraphenylenediamine (PPD), a common allergen found in dark hair dyes and is an adulterant in henna used for tattoos. It is also an antioxidant used in the production of rubber.^1^ Although PPD allergic reactions are well described in the literature, published images in darker skin tones are rare.

## Case report

**Fig 1:**
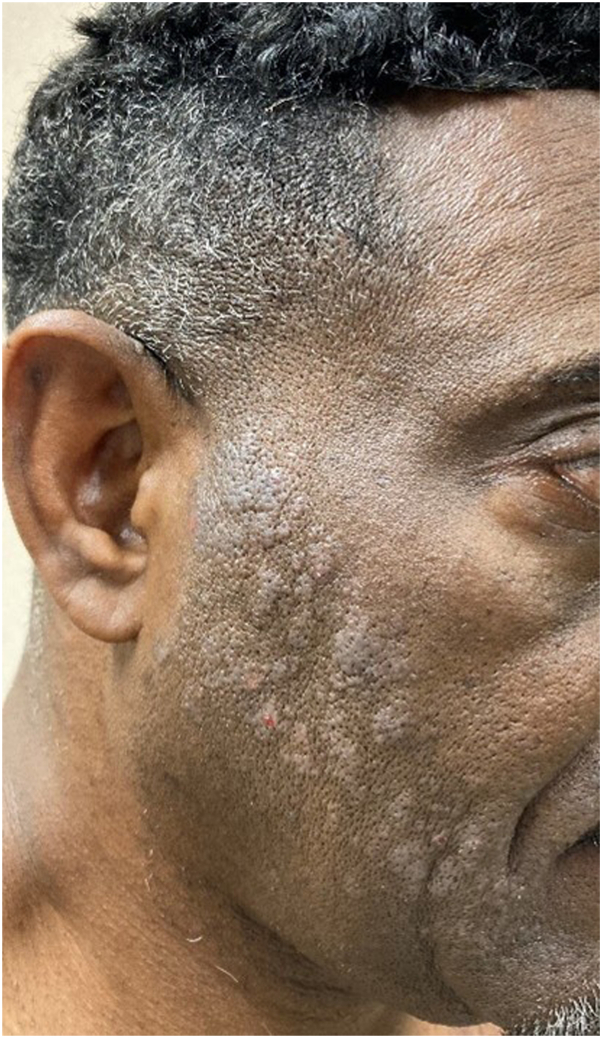
The patient presented with slightly hypopigmented, 1 to 15 mm mildly scaly, and focally excoriated, papules on the cheeks, postauricular area and back of the neck. These developed within 48 hours after applying black hair dye containing paraphenylenediamine. The dramatic erythema associated with contact dermatitis in light skinned patients is subtle in this patient with type V skin.

A 57-year-old African American male with human immunodeficiency virus presented with a pruritic, patchy rash on the face, neck, and chest that appeared 2 days after application of a hair dye containing PPD. A facial rash occurred once before within 24 to 48 hours after application, but a relationship to hair dye was discounted by the patient since the scalp was not involved. Medications included bictegravir-emtricitabine-tenofovir and hydrochlorothiazide. Scaling, edematous, focally excoriated, hypopigmented, and slightly erythematous papules were present on the cheeks, postauricular areas, and back of the neck (Fig 1). The scalp was spared. Treatment was initiated with desonide 0.05% cream twice daily for 1 week and tapered with significant improvement. A single patch test with 1% PPD was applied to the right forearm producing mild pruritus with erythema and tiny papules at the patch site at 48 hours. A 6-day reading demonstrated a strong 3+ positive reaction with erosions.

## Discussion

Patients rarely consider the diagnosis of contact dermatitis to hair coloring because the scalp beneath the dyed hair, where they would expect a reaction to occur, is resistant to allergic contact dermatitis. This resistance has been attributed to a high concentration of T regulatory cells surrounding hair follicles. These cells play an important role in maintaining immune homeostasis.^2^ Dermatologists can also miss the diagnosis of PPD-induced allergic contact dermatitis especially in darker skin types since erythema is obscured.^3^, ^4^, ^5^ We reviewed over 2000 publications and found images of only 3 cases of PPD sensitivity in patient with skin type V (see firstness study in Supplementary Material, available via Mendeley at https://data.mendeley.com/datasets/65xwbxyyyz/1). In addition, African American patients may have more limited access to specialized dermatologic care.

It is also notable that medications with a para-amino structure, such as hydrochlorothiazide, furosemide, sulfamethoxazole, and/or benzocaine, may cross-react to PPD.^6^ This is particularly important since hydrochlorothiazide is a preferred initial monotherapy in hypertensive African American patients.^7^ In fact, our patient was taking hydrochlorothiazide. Finally, patients allergic to PPD often have negative patch test readings at the time of the first reading on day 2. Late readings, usually at day 7, are required to identify positive patch tests for a variety of allergens, including PPD, multiple metals, some preservatives, acrylates, and neomycin.^8^, ^9^, ^10^

For these patients, it is critical to avoid contact with black hair dye containing PPD. Fortunately, PPD-free dyes and vegetable-based hair dyes are generally safe in PPD sensitized patients. These alternatives, however, should be used cautiously since compounds such as para-toluenediamine sulfates may be present and cross-react in 43% of individuals who are sensitive to PPD.^1^ In summary, a patient with a facial rash preceded by application of hair dye should be patch tested. PPD sensitivity responds to short courses of topical steroid and systemic steroids (10-14 days) and avoidance of the allergen.

## Conflicts of interest

Dr Brodell is a principal investigator for a clinical trial sponsored by Novartis, the CorEvitas Psoriasis Biologic registry, and owns stock in Veradermics, Inc. He serves on editorial boards of American Medical Student Research (faculty adviser), Practice Update Dermatology (Editor-in-Chief), Journal of the American Journal Academy of Dermatology (Associate Editor), Practical Dermatology, Journal of the Mississippi State Medical Society, SKIN: The Journal of Cutaneous Medicine, and Archives of Dermatological Research. Authors McClain and Brown and Drs Noble and Helms have no conflicts of interest to declare.
